# Reactive Balance Control in Response to Perturbation in Unilateral Stance: Interaction Effects of Direction, Displacement and Velocity on Compensatory Neuromuscular and Kinematic Responses

**DOI:** 10.1371/journal.pone.0144529

**Published:** 2015-12-17

**Authors:** Kathrin Freyler, Albert Gollhofer, Ralf Colin, Uli Brüderlin, Ramona Ritzmann

**Affiliations:** 1 Department of Sport and Sport Science, Albert-Ludwigs-University Freiburg, Freiburg, Germany; 2 Department of Mechatronics, University of Applied Science, Esslingen, Germany; University of Rome Foro Italico, ITALY

## Abstract

Unexpected sudden perturbations challenge postural equilibrium and require reactive compensation. This study aimed to assess interaction effects of the direction, displacement and velocity of perturbations on electromyographic (EMG) activity, centre of pressure (COP) displacement and joint kinematics to detect neuromuscular characteristics (phasic and segmental) and kinematic strategies of compensatory reactions in an unilateral balance paradigm. In 20 subjects, COP displacement and velocity, ankle, knee and hip joint excursions and EMG during short (SLR), medium (MLR) and long latency response (LLR) of four shank and five thigh muscles were analysed during random surface translations varying in direction (anterior-posterior (sagittal plane), medial-lateral (frontal plane)), displacement (2 vs. 3cm) and velocity (0.11 vs. 0.18m/s) of perturbation when balancing on one leg on a movable platform. *Phases*: SLR and MLR were scaled to increased velocity (*P*<0.05); LLR was scaled to increased displacement (*P<0*.*05*). *Segments*: phasic interrelationships were accompanied by segmental distinctions: distal muscles were used for fast compensation in SLR (*P<0*.*05*) and proximal muscles to stabilise in LLR (*P<0*.*05*). *Kinematics*: ankle joints compensated for both increasing displacement and velocity in all directions (*P<0*.*05*), whereas knee joint deflections were particularly sensitive to increasing displacement in the sagittal (*P<0*.*05*) and hip joint deflections to increasing velocity in the frontal plane (*P<0*.*05*). COP measures increased with increasing perturbation velocity and displacement (*P<0*.*05*). Interaction effects indicate that compensatory responses are based on complex processes, including different postural strategies characterised by phasic and segmental specifications, precisely adjusted to the type of balance disturbance. To regain balance after surface translation, muscles of the distal segment govern the quick regain of equilibrium; the muscles of the proximal limb serve as delayed stabilisers after a balance disturbance. Further, a kinematic distinction regarding the compensation for balance disturbance indicated different plane- and segment-specific sensitivities with respect to the determinants displacement and velocity.

## Introduction

In balance research, the setup of a translating platform via externally applied perturbations is used to investigate underlying control mechanisms of compensatory balance responses in standardised laboratory conditions [1–4]. Previous studies examining reactive balance control in response to perturbation have mainly been executed in bipedal paradigms and it is quite well described how postural stability is controlled with double limb support when unidirectional surface translations are induced in predictable experimental settings with constant perturbation parameters [2,3,5–8]. However, there is very little knowledge of how individuals recover balance under single leg stance conditions, despite the fact that losses of balance often occur under these conditions [9–11]. Moreover, usually various interdependent variables—i.e., unpredictable magnitude, velocity or direction of stimulus origin—challenge postural equilibrium simultaneously. Consequently, the composition of the stimulus strongly influences compensatory responses [12]. To gain a thorough understanding of the mechanisms contributing to re-stabilisation after perturbation, the complex mechanisms of regaining postural stability in an unilateral stance paradigm must also be part of balance research. Further, the interdependence of various stimulus characteristics should be taken into account, as the interactions of different perturbation variables may have specific effects on the modulation of the postural response [5,13,14].

From studies examining compensatory neuromuscular responses after perturbations in static paradigms, mainly executed in the 1980s and 1990s (see review [15]), it is known that the central nervous system plays a crucial role in governing appropriate muscle forces to prevent falling by relocating the centre of gravity (COG). It has been shown that muscular activation patterns are characterised by phase-specific reflex components indicated as short (SLR), medium (MLR) and long (LLR) latency responses following the onset of perturbation [5,8,16]. Dietz et al. [2,3,7] and Gollhofer [17] demonstrated that functionally relevant muscle activation (> 65ms after onset, MLR and LLR) occurs when the COG is shifted away from the vertical. MLR and LLR are supposed to be attributed to spinal, polysynaptic reflexes and have functional significance to induce appropriate active joint moments for the preservation of postural stability [2,3,8,12,17]. Slight postural disturbances (mainly small rotations around the ankle joint, less visible translation) are compensated by immediate, non-functional monosynaptic stretch responses in the SLR [16,17].

In view of reflexive muscle compensation in response to surface translation, distinction is drawn between perturbation direction, displacement and velocity; experiments executed during bilateral stance indicate that early, monosynaptic stretch responses are sensitive to perturbation velocity [16], whereas the later functional components of muscle activation patterns were demonstrated to compensate for alterations in the displacement of platform translation [5,6]. Further, it is suggested that, by controlling a multi-segment system, neuromuscular control after balance perturbation includes a segmental distribution of compensatory electromyographic (EMG) responses [4,12,18]. Authors speculate that mechanical coupling of sensory inputs at ankle, knee and hip joints induce corresponding activation of distal and proximal limb muscles [4,12], but the functional pattern of interlimb activation during stabilisation is still unclear. Further, researchers showed that the direction of surface translation is also of considerable importance for the output of the postural response. Although less examined, there is evidence that muscles play different functions as stabilisers during the postural response, and it has been demonstrated that most muscles primarily act in one direction, independently of measurement condition [19,20]. Apparently, Moore and colleagues [19] discovered that predominantly the distal muscles act in one direction, whereas proximal muscles cover a greater range of trajectories in the horizontal plane.

Based on these aspects, it is assumed that postural responses are modified according to functional requirements of a stable equilibrium, and different phasic and segmental strategies are used depending on perturbation characteristics to respond quickly and accurately to the balance disturbance [5,12,16].

**Fig 1:**
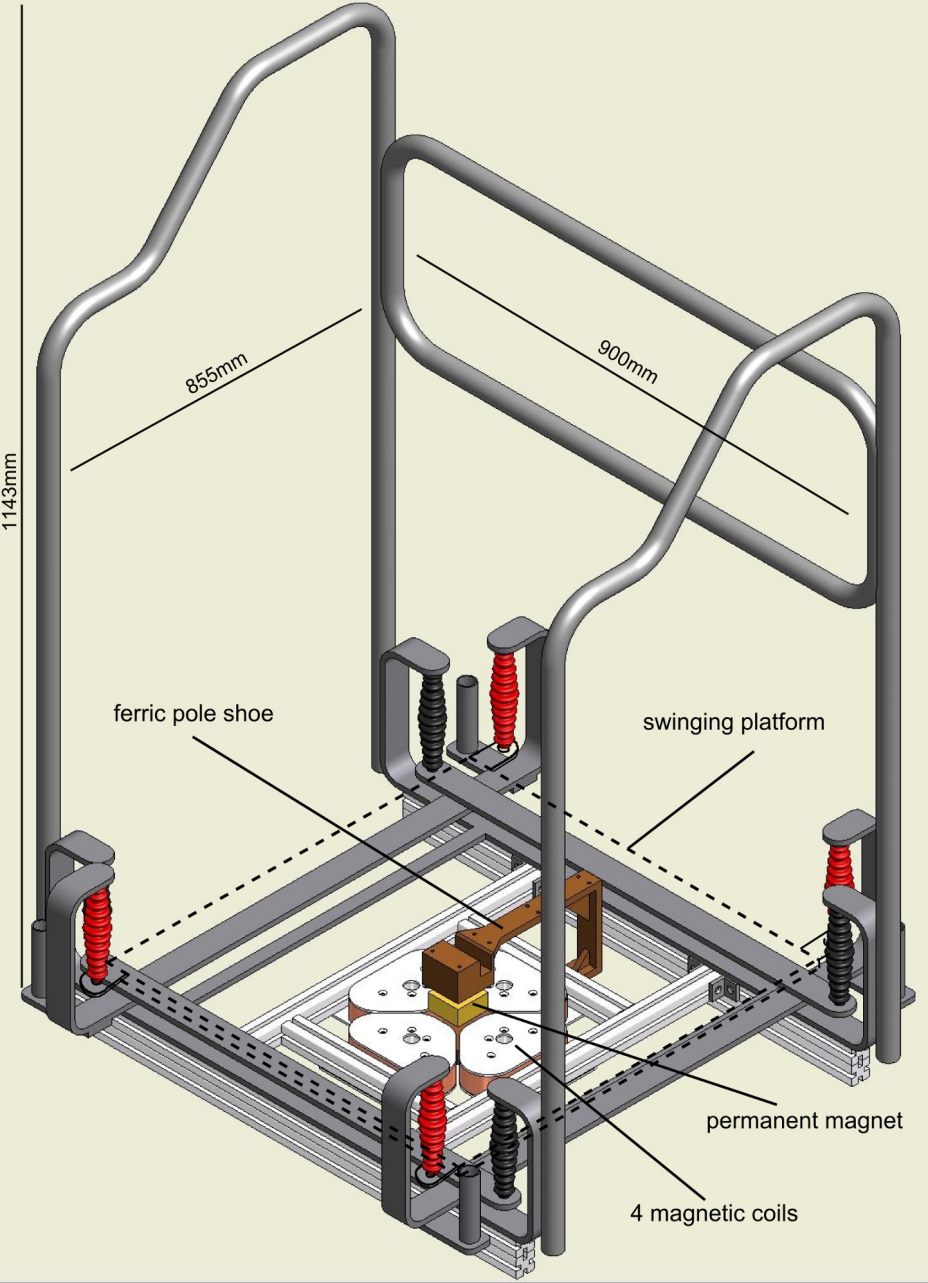
Hardware construction of the electromagnetically driven perturbation platform (Perturmed^®^). The basic hardware of the Perturmed^®^ consists of an already existing device, the Posturomed^®^ [23–25]. The Perturmed^®^ construction comprises a freely swinging platform (dashed line, 40x40cm) which is fixed with eight steel ropes (red and black): the platform itself is attached to four steel ropes (red), they in turn are attached to another iron frame hanging freely on the other four steel ropes (black). Thus, the freely swinging support surface is in total attached to a solid iron frame via two steel ropes on each corner. The pole shoe with the permanent magnet is fixed beneath the platform; the four magnetic coils are attached below at the bottom of the iron frame. By activation of two opposed interconnected coils via temporal current feed they release attracting and repelling electromagnetic forces, which move the support surface into the respective direction. The safety construction consisting of a solid metallic frame was used to secure the subjects from falling; fall rate within this experiment was below 2%. For the measurements, the electromagnetic forces were used to apply unpredictable horizontal translations of the free-swinging platform, gradually adjustable in direction, displacement and velocity. Platform kinematics were controlled by means of a movable goniometer attached to the platform.

**Fig 2:**
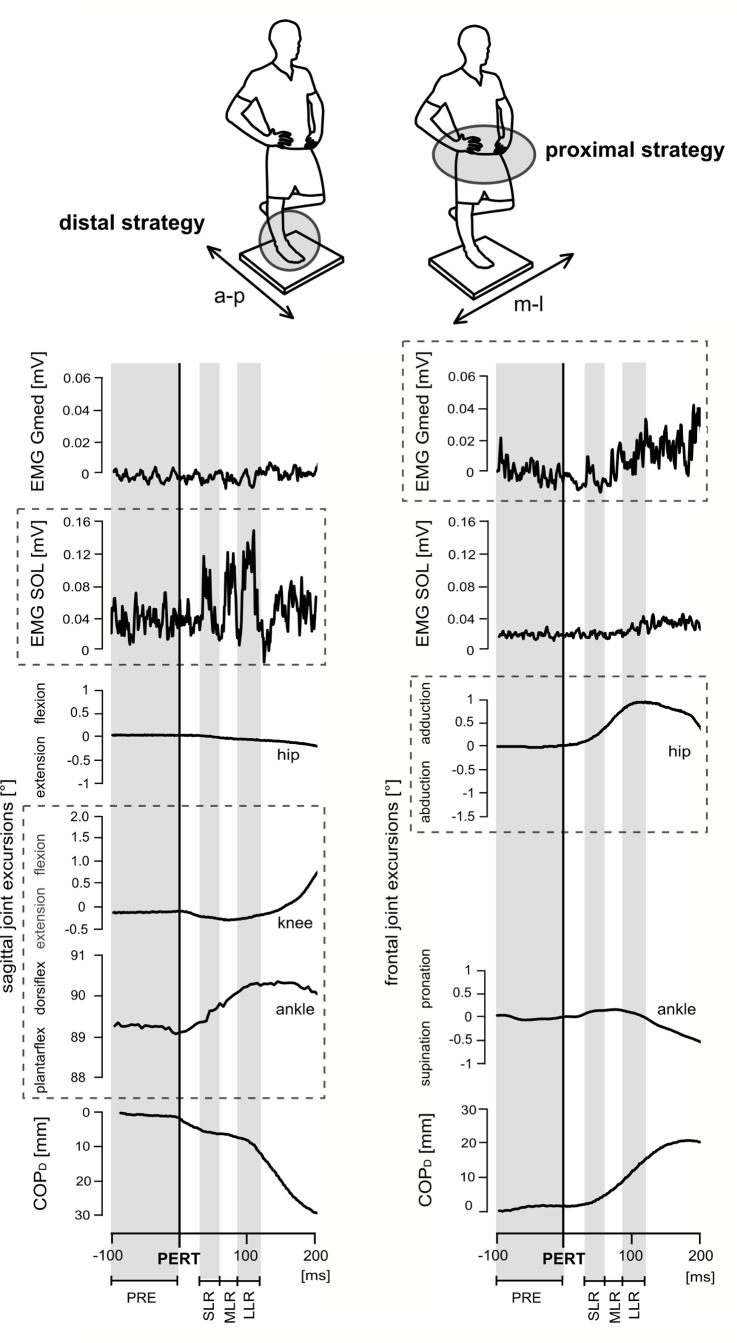
Neuromuscular and kinematic responses of one subject (top) during posterior (left) and lateral (right) perturbation. The top shows the subject standing on his right leg on the support surface (40x40cm), head and eyes directed forward, right knee joint extended and hands on the hips as required for the 30s measurement period. The support surface was a freely swinging platform. Thus, subjects needed to stabilise equilibrium even without perturbation. Below, the time point of perturbation (PERT) is marked as the black line; highlighted with grey/white backgrounds are the different temporal phases of the recorded measurements (PRE, SLR, MLR, LLR). Perturbations in the sagittal plane cause postural reactions in distal muscles accompanied by ankle and knee joint deflections referring to the ankle strategy using the distal segment for compensation, whereas during perturbations in the frontal plane, mainly hip joint deflections counteract balance disturbance by using proximal muscles, indicating the use of the hip strategy (visualised by the dashed boxes).

The present study provides a new approach in balance research comprising an unilateral balance design with neuromuscular activity and kinematics expressed as a function of three interrelated perturbation determinants direction, displacement and velocity. In order to achieve a more comprehensive understanding of the neuro-mechanical coupling during unilateral balance control, for the first time, random perturbations were applied while balancing in an unstable unilateral stance. To provide unstable balance conditions, the experimental setting comprised a freely swinging platform which was perturbed in each direction in the horizontal plane (Fig 1). Therefore, the purpose of the study was to investigate the interaction effects of three randomly varied determinants (direction, displacement and velocity) and their influence on the phase-specific EMG pattern and segmental regulation of leg muscle activation as well as on the joint deflections and centre of pressure (COP) displacement during perturbed, unilateral stance (Fig 2). We executed the experiments regardless of acceleration and deceleration profiles. We hypothesised that study would reveal interaction effects for the variables direction, displacement and velocity. We further hypothesised that those determinant-dependent modulations would be phase (SLR, MLR and LLR) and segment specific (distal and proximal), and may be associated with differences in the selected balance strategy, accompanied by distinctions in kinematic output, depending on the combination of the variables. We expected that the higher the magnitude of displacement and velocity, the higher the neuromuscular and kinematic postural responses and the more proximal those responses would occur.

## Methods

### Subjects

Based on the results of a pilot study including five subjects, a power analysis (f = 0.4; alpha = 0.05; power = 0.9 for ANOVA) revealed that a participation of 20 volunteers is needed in this study. The participants were physically fit students in the department of sports and sports science, with no previous neurological irregularities or injuries to the lower extremities (6 women and 14 men, age 27±3years, weight 73±12kg, height 178±9cm; variables are expressed as mean±standard deviation). All subjects provided written informed consent for the experiment, which was approved by the ethics committee of the University of Freiburg, and was in accordance with the latest revision of the Declaration of Helsinki.

### Experimental design

**Fig 3:**
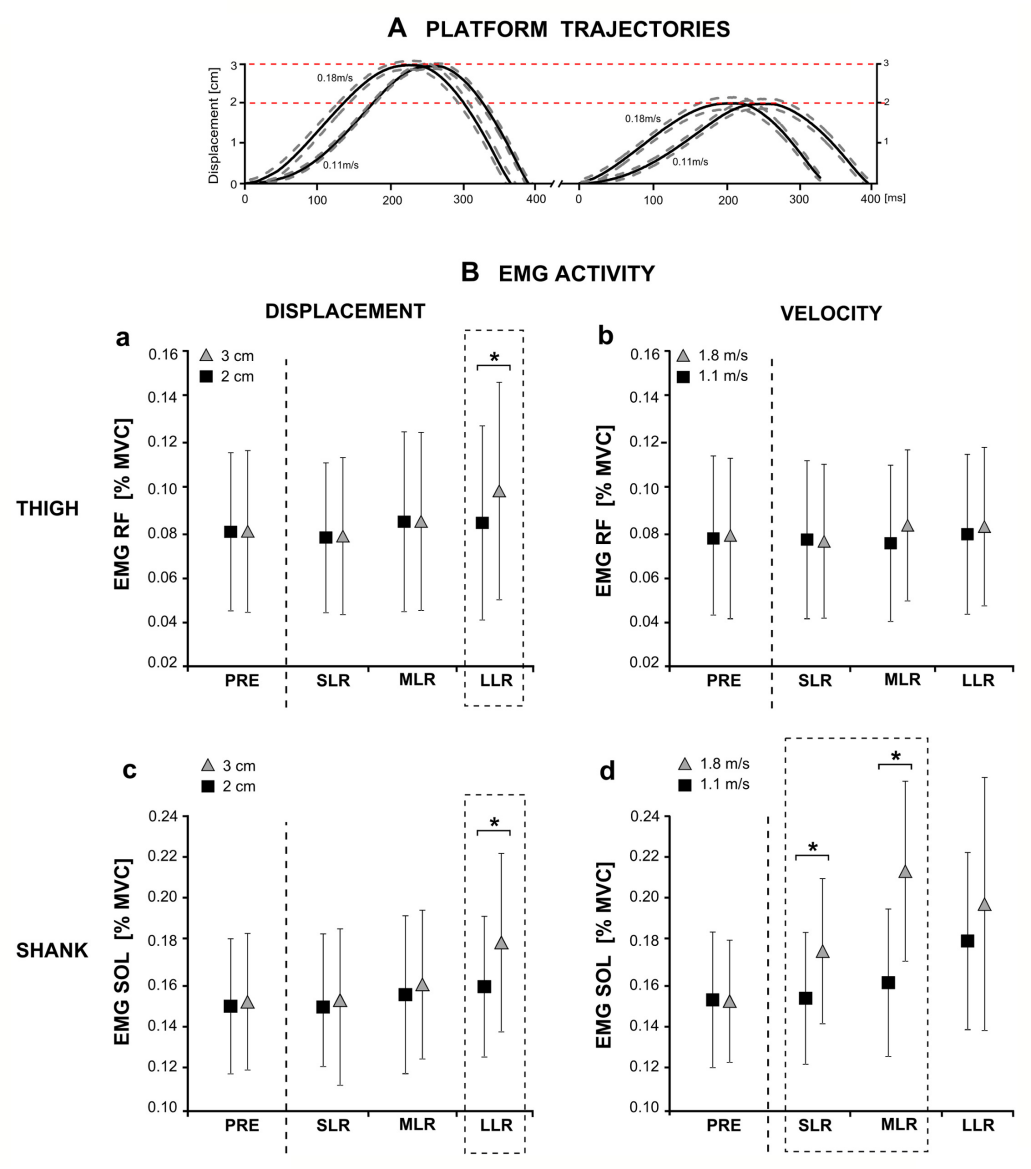
(A) Platform trajectories for perturbation displacements and velocities and (B) corresponding modulations in neuromuscular responses. (A) Grand means of platform trajectories that illustrate pre-setting for displacement and velocity. (B) Mean changes in neuromuscular activity of all subjects in one representative thigh (a & b) and shank (c & d) muscle in response to increased perturbation displacement (a & c) and velocity (b & d) during the temporal phases before (PRE) and after (SLR, MLR, LLR) the perturbation (separated by dashed line). The phase- and segment-specific interaction effects are elucidated by the dashed boxes: muscles of the shank and thigh are used to compensate for increased perturbation displacement (grey triangle), and the neuromuscular response occurs in LLR (a & c). In contrast, only shank muscles are used for a fast compensation of increased perturbation velocity (grey triangle) during the early reflex phases SLR and MLR (b & d). * indicates a significant difference for pairwise comparisons (p<0.05).

A single-group repeated-measures crossed study design was used to examine the influence of three perturbation-related determinants on neuromuscular activity, joint kinematics and COP displacement during a monopedal stance (Fig 2). Unilateral stance was preferred to bipedal stance as it is more relevant in fall situations due to a smaller support surface [9–11]. For that purpose, the EMG activity of four shank and five thigh muscles, the displacement and velocity of the COP as well as the joint excursions in the sagittal (ankle, knee and hip) and frontal (ankle and hip) planes were analysed with respect to the direction (anterior-posterior vs. medial-lateral [20]), the displacement (2 vs. 3cm [21], Fig 3A) and the velocity (0.11 vs. 0.18m/s [5,22], Fig 3A) of randomly applied perturbations. Directions, displacements and velocities were chosen according to previous experiments executed in bilateral stance conditions [5,20–22] and consequently parameter reliability had been tested in a pilot study including 15 subjects.

### Platform construction

Perturbations were generated by means of the Perturmed^®^ (Brüderlin, Germany). The basis of this construction is an already existing device (Posturomed^®^, Haider Bioswing, Germany) with a high test-retest reproducibility, which consists of a platform attached to a solid frame via two steel ropes on each corner [23–25] (see Fig 1). To move the platform reliably, electromagnetic forces were used to apply unpredictable horizontal translations of the free-swinging platform by means of coils affixed beneath the frame and a permanent magnet attached below the platform (Fig 1). Platform trajectories (displacement and velocity) were controlled by means of a movable goniometer attached to the platform and synchronised to the neuromuscular and kinematic recordings (grand means are illustrated in Fig 3A). The three perturbation determinants and their different characteristics were applied stochastically (16 combinations: four directions x two displacements x two velocities). Altogether, 20 perturbations for each combination were collected per subject. To control for the reliability of the subject’s starting position, trials were eliminated when platform trajectories prior to or after perturbation were beyond ±0.2cm (equivalent to 1.33% of the whole platform range).

### Test procedure

Prior to measurements, subjects participated in familiarisation sessions for 10 minutes to adapt to the unstable surface and the perturbation mechanism in order to eliminate learning effects within the measurements. During testing, the subjects stood barefoot in an upright position on their right leg, kept hands on the hip and directed their head and eyes forward. They were instructed to stand as still as possible, with the unsupported leg flexed at 45° and not touching the other leg. Foot placement on the platform was controlled by means of a stencil to keep the subjects’ feet in the same starting position for all trials. To control for the reliability of the subject’s starting position, the body position of the standing leg was controlled by goniometers and two operators. Perturbations were applied randomly every 2–4 seconds in sets of 10 perturbations separated by a minimum of 30 seconds of rest in between for recovery [19,21,26]. Subjects were instructed to stabilise equilibrium as quickly as possible; in case of struggling or falling—defined as attempts where subjects failed to regain postural equilibrium after surface translation and i) touched the safety frame of the Perturmed^**®**^ with at least one hand or ii) touched the ground with the unsupported (left) foot to avoid falling—perturbations were repeated.

For normalisation of the EMG data, prior to the measurements subjects performed three isometric maximal voluntary contractions (MVC) for each recorded muscle; we used the trial with the highest EMG for data normalization. The MVCs were executed according to [27] and [28], performed isometrically against resistance and held for three seconds. Between trials and repetitions subjects had recovery pauses of one minute. Body position during MVCs was strictly controlled and standardized by means of supervision by the authors and by goniometric recordings of ankle, knee and hip joint angles. Antagonistic muscle activation was monitored and trials repeated when antagonists were activated.

### Dependent variables

The variables EMG data of nine muscles, COP movement, platform trajectories and joint kinematics were synchronously recorded using a signal (5V, 1ms width) triggered to occur at the instant of platform perturbation. During perturbations, subjects stood on their right leg.

#### EMG recording

EMG data were obtained by placing bipolar surface electrodes (⊘9mm, Ag/AgCl, Ambu Blue Sensor P, Ballerup, Denmark) over the m. soleus (SOL), gastrocnemius medialis (GM), tibialis anterior (TA), peroneus longus (PER), rectus femoris (RF), vastus lateralis (VL), biceps femoris (BF), gluteus medius (Gmed) and gluteus maximus (Gmax) of the right leg. Electrodes were placed in line with the direction of the underlying muscle fibres with a centre-to-centre distance of 25mm according to SENIAM guidelines [29]. By shaving and light abrasion of the skin, interelectrode resistance was kept below 2.5kΩ. Signals were amplified (x1000) and recorded with 1kHz (band-pass filter 10Hz–1kHz).

#### Postural sway

COP displacement prior to and following perturbation was monitored by means of a pressure distribution measuring system (pedar^®^, Novel, Germany, [30]). The sensor mat was placed upon the platform; COP was recorded with 100Hz sampling rate and a spatial resolution of four sensors per square centimeter. Subsequently, peak COP displacement (COP_D_) and velocity (COP_V_) were calculated. COP assessment was executed by 3D sensor deformation technology (500g) instead of using a force plate, as the force plate would have enlarged the mass and consequently the inertia of the swinging component of the Perturmed^®^. To standardize the subject’s starting position and consequently to control for the subject’s forward or backward shifts [31] as well as any shift to the laterals left or right, trials were eliminated when COP trajectories prior to perturbation were beyond ±0.2cm.

#### Joint kinematics

Ankle, knee and hip joint excursions in the sagittal plane in response to anterior and posterior (a-p) perturbations, as well as ankle and hip joint excursions in the frontal plane in response to medial and lateral (m-l) perturbations were recorded with electro-goniometers (Biometrics®, Gwent, UK) consisting of a centre of rotation and two movable endplates. The endplates have a length of 10cm each and a range of 270°. The centre of rotation was placed over the respective joint centre; each endplate was attached to the prolonged axis of the anatomical structures [32].

Sagittal plane: the centre of rotation was fixed over the lateral malleolus (ankle), over the knee joint cavity (knee) and over the Trochanter major (hip). The two endplates were aligned pointing towards the fifth metatarsal and longitudinal axis of the shank (ankle), towards the lateral malleolus and Trochanter major (knee) and towards the longitudinal axis of the femur and thorax (hip). 90° between the fifth metatarsal and the fibula was defined as a 90° ankle angle; plantar flexion was reflected by an angle greater than 90°. The knee and hip flexion angle was set to zero at 0° during an upright stance, and joint flexion was reflected by an angle greater than 0°.

Frontal plane: the centre of rotation was fixed over the heel (ankle) and over the frontal Trochanter major (hip); the two endplates were aligned pointing towards the upper Achilles tendon and the Calcaneus (ankle), and towards the longitudinal axis of the femur and the abdominal wall (hip). The ankle and hip flexion angle was set to zero at 0° during an upright stance; lateral joint flexion was reflected by an angle greater than 0°, medial joint flexion by an angle smaller than 0°. Signals were recorded with 1kHz and filtered (10Hz–1kHz).

### Data processing

Each perturbation was analysed in a 500ms interval, comprising 100ms prior to and 400ms after perturbation onset (-100 to 400ms).

EMG during MVC was integrated for each muscle for a time frame of one minute [mVs]; the trial with the highest EMG was used for normalization.

We analysed one perturbation direction for each of the muscles; i.e. the EMG responses of muscles which are mainly used to counteracted surface translation in the respective perturbation direction [20]. For each muscle, integrated EMGs (iEMG) were calculated. For data analysis, iEMG was divided into four relevant phases: the pre-activation phase 100ms prior onset of perturbation (PRE, -100–0ms) and three compensatory postural responses based on the latencies of the reflex phases. Those are defined as follows: the short latency response from 30ms after onset of perturbation until 60ms (SLR, 30–60ms [33]), the medium latency response (MLR, 60–85ms [26]) and the late latency response (LLR, 85–120ms [26]). Subsequently, iEMGs were time normalised [mV/s] for the comparability of iEMGs between phases, then normalised to the respective MVC [%MVC] and averaged for subjects and perturbation conditions.

Ankle, knee and hip joint kinematics were expressed as mean angular displacement [°] for each subject and each perturbation condition, and were calculated as the difference between the peak angle position (defined as the maximum value of the angle excursion within the 400ms window) and the onset position.

COP_D_ [mm] was calculated for each subject and perturbation condition as the difference between the peak COP position (defined and marked manually as the maximum value of the COP excursion within the 400ms window) and the onset position. COP_V_ was calculated according to [26]: COP_V_ [mm/ms] = COP_D_/t (with t defined as the time interval from the start of perturbation to the time point of the peak).

### Statistics

To analyse the effects of the three perturbation determinants (direction, displacement and velocity) on the respective neuromuscular and kinematic variables, and to detect interaction effects between the independent variables, within-subject comparisons were performed using a repeated measures analysis of variance (ANOVA). To evaluate the responses of the COP measures (COP_D_ and COP_V_) and ankle, knee and hip joint excursions, a three-factor ANOVA was used, respectively [direction (4) x displacement (2) x velocity (2)].

To assess the neuromuscular responses according to varying displacement and velocity, a two-factor ANOVA was calculated for SLR, MLR and LLR, respectively [displacement (2) x velocity (2)]. The level of significance was set at P<0.05. To correct for multiple testing we used Bonferroni correction; each P-value (P_i_) for each test was multiplied by the number of tests (P_i adjusted_ = P_i_ * n, n = number of tests). If P_i adjusted_ was <0.05 we considered the respective test *i* to be of statistical significance. If the assumption of sphericity measured by Mauchly's sphericity test was violated, the Greenhouse-Geisser correction was used. In case of significant main effects, post hoc comparisons (Tukey’s HSD, level of significance *p*<0.05) were calculated for specification of the direction of the particular differences. Analyses were executed by using SPSS 20.0 (SPSS, Inc., Chicago, IL, USA).

## Results

In Table 1, mean values of the COP_D_, COP_V_ as well as ankle, knee and hip joint excursions are displayed for the different perturbation conditions. The EMG activity of the nine leg muscles during the four phases (PRE, SLR, MLR, LLR) are shown in Table 2.

**Table 1:** Changes of the COD_D_, COP_V_ and ankle, knee and hip joint deflections with respect to the different permutations of perturbation direction, velocity and displacement are illustrated.

direction	anterior				posterior				medial				lateral			
velocity	0.11m/s		0.18m/s		0.11m/s		0.18m/s		0.11m/s		0.18m/s		0.11m/s		0.18m/s	
displacement	2cm	3cm	2cm	3cm	2cm	3cm	2cm	3cm	2cm	3cm	2cm	3cm	2cm	3cm	2cm	3cm
**COPd [mm]**	16.6±3.7#	23.5±5.1*#	13.2±3.5†#	22.1±5.6†*#	16.6±3.2#	24.7±4.9*#	14.5±2.8†#	25.6±4.0†*#	13.0±2.9#	15.8±3.0*#	11.3±2.0†#	15.9±2.8†*#	13.4±2.5#	16.3±2.9*#	11.6±1.8†*#	15.5±2.7 †*#
**COPv[mm/s]**	0.8±0.2 #	1.2±0.2 *#	0.9±0.3 †#	1.4±0.3 †*#	0.7±0.1 #	1.0±0.2 *#	1.0±0.2 †#	1.4±0.3 †*#	0.4±0.1 #	0.6±0.1 *#	0.5±0.1 †#	0.7±0.1 †*#	0.5±0.1 #	0.6±0.1 *#	0.5±0.1 †*#	0.7±0.1 †*#
**Joint exc. [°]**	**sagittal**								**frontal**							
ankle	0.8±0.7	1.2±1.1 *	0.7±0.6 †	1.1±1.0 †*	0.9±0.8	1.2±1.2 *	0.6±0.5 †	0.8±0.8 †*	2.6±1.6 #	3.5±2.0 *#	2.0±1.4 †#	3.7±2.1 †*#	1.7±1.2 #	2.1±1.5 *#	1.2±0.7 †*#	1.7±1.0 †*#
knee	1.3±0.4	2.1±0.5 *	0.9±0.4 †	2.0±0.6 †*	1.0±0.5 #	1.6±0.6 *#	0.6±0.3 †#	1.3±0.5 †*#								
hip	0.5±0.2	0.8±0.3 *	0.4±0.2 †	0.7±0.3 †*	0.4±0.2	0.8±0.3 *	0.3±0.1 †	0.6±0.2 †*	0.7±0.5	1.1±0.7 *	0.6±.05 †	1.3±0.9 †*	0.8±0.4	1.3±0.6 *	0.5±0.3 †*	0.9±0.4 †*

Values represent mean values ± standard deviations (M±SD). For significant main effects of the ANOVA, a * symbol marks a significant effect of the displacement on the respective parameters measured, a † symbol marks a significant effect of the velocity and a # symbol marks a significant effect of the perturbation direction (*P<0*.*05*).

**Table 2:** Modulations in EMG activity in the time interval before (PRE) and the three reflex phases after perturbation (SLR, MLR, LLR) according to the different perturbation velocities and displacements.

EMG [%MVC]	PRE				SLR				MLR				LLR			
velocity	0.11m/s		0.18m/s		0.11m/s		0.18m/s		0.11m/s		0.18m/s		0.11m/s		0.18m/s	
displacement	2cm	3cm	2cm	3cm	2cm	3cm	2cm	3cm	2cm	3cm	2cm	3cm	2cm	3cm	2cm	3cm
**SOL** posterior	0.15±0.03	0.15±0.03	0.15±0.03	0.15±0.03	**0.15±0.04**	**0.15±0.03**	**0.16±0.03 †**	0.18±0.03 †*	***0*.*16±0*.*04***	***0*.*16±0*.*04***	***0*.*18±0*.*04 †***	***0*.*21±0*.*04*** *†	*0*.*16±0*.*03*	*0*.*18±0*.04 *	*0*.*17±0*.*04*	*0*.*20±0*.06 *
**GM** posterior	0.18±0.07	0.17±0.07	0.17±0.07	0.17±0.07	**0.17±0.07**	**0.16±0.08**	**0.20±0.10**	**0.17±0.08** *	**0.17±0.07**	**0.17±0.08**	**0.38±0.14 †**	**0.43±0.16 †**	*0*.*22±0*.*09*	*0*.*31±0*.12 *	*0*.*36±0*.*11 †*	*0*.*54±0*.13 †*
**TA** anterior	0.14±0.08	0.14±0.08	0.14±0.08	0.13±0.08	0.14±0.08	0.14±0.08	0.14±0.09	0.18±0.21	**0.14±0.09**	**0.13±0.07**	**0.19±0.13**	**0.20±0.11 †**	*0*.*16±0*.*10*	*0*.*16±0*.*10*	*0*.*24±0*.*11 †*	*0*.*38±0*.19 †*
**PER** lateral	0.26±0.12	0.27±0.12	0.26±0.13	0.25±0.12	**0.26±0.12**	**0.27±0.13**	**0.34±0.20†**	**0.29±0.15**	**0.24±0.09**	**0.23±0.08**	**0.45±0.16 †**	**0.43±0.17 †**	*0*.*34±0*.*15*	*0*.*43±0*.*14*	*0*.*61±0*.*19 †*	*0*.*76±0*.25 †*
**RF** medial	0.08±0.04	0.08±0.03	0.08±0.03	0.08±0.04	0.08±0.03	0.08±0.03	0.08±0.03	0.08±0.03	0.08±0.03	0.08±0.03	0.08±0.04	0.08±0.04	0.08±0.04	0.08±0.03	0.08±0.04	0.10±0.05
**VL** anterior	0.12±0.06	0.12±0.06	0.12±0.06	0.12±0.06	0.12±0.06	0.12±0.06	0.12±0.06	0.12±0.06	0.12±0.06	0.12±0.06	0.15±0.08 †	0.16±0.08 †	*0*.*13±0*.*06*	*0*.*14±0*.*07*	*0*.*15±0*.*09*	*0*.*19±0*.12 †*
**BF** posterior	0.25±0.11	0.25±0.11	0.25±0.11	0.25±0.12	0.24±0.11	0.23±0.11	0.24±0.11	0.24±0.11	0.23±0.11	0.23±0.11	0.25±0.12	0.25±0.12	0.25±0.12	0.25±0.11	0.27±0.14	0.30±0.19
**Gmed** medial	0.29±0.10	0.30±0.10	0.28±0.12	0.30±0.11	**0.29±0.11**	**0.28±0.11**	**0.27±0.10†**	**0.27±0.10**	*0*.*28±0*.*11*	*0*.*27±0*.*10*	*0*.*29±0*.*11*	*0*.*29±0*.12 *	0.29±0.11	0.29±0.11	0.31±0.13	0.30±0.13
**Gmax** posterior	0.19±0.08	0.19±0.08	0.19±0.08	0.19±0.08	0.18±0.08	0.18±0.07	0.19±0.07	0.18±0.08	0.18±0.07	0.18±0.08	0.18±0.07	0.19±0.08	0.18±0.08	0.18±0.07	0.19±0.08	0.19±0.08

Muscles are displayed for the respective perturbation direction, in which they counteracted surface translation. The table elucidates that dominant changes to increased displacement only occurred within the fast velocity. Moreover, increased perturbation velocity was predominantly compensated in SLR and MLR (bold font), whereas increased displacement was predominantly compensated in LLR (italic font). Values represent mean values ± standard deviations (M±SD). For significant main effects of the ANOVA (*P<0*.*05*), the lines written in italic font denote a significant effect of the displacement on the specific phase (a * symbol marks significant post hoc comparison, p<0.05) and the lines written in bold font denote a significant effect of the velocity (a † symbol marks significant post hoc comparison, p<0.05). The line written in bold and italic font contains both effects (displacement and velocity) on the specific phase.

### Direction of the perturbation

EMG responses were analysed in the direction in which they were maximally active: for posterior direction SOL, GM, BF and Gmax; for anterior direction TA and VL; for medial direction Gmed and RF and for lateral direction PER (Table 2).

The factor direction had a significant main effect on COP_D_ (P<0.001, F = 66.95), COP_V_ (P<0.001, F = 129.96) as well as on ankle joint deflection in the frontal plane (P = 0.02, F = 18.41) and knee joint deflection in the sagittal plane (P<0.001, F = 39.11). For all conditions, COP_D_ shifted contrarily to the perturbation direction, i.e. a forward translation of the platform caused a backwards shift of the COP.

Ankle and knee joint excursions deflected according to each perturbation direction (in the sagittal plane for a-p, in the frontal plane for m-l perturbations, Fig 2). Perturbation direction had no influence on hip joint excursion.

### Displacement of the perturbation

Displacement-induced changes in EMG activity occurred primarily in LLR: with increasing displacement, EMG responses in GM (P<0.001; F = 89.46), TA (P<0.001; F = 34.59), PER (P = 0.01; F = 25.57) and VL (P = 0.04; F = 11.48) increased in LLR only (Fig 3B). SOL EMG was enhanced in MLR (P = 0.01; F = 18.43) and LLR (P = 0.01; F = 18.47), Gmed in MLR (P<0.001; F = 37.98).

COP_D_ (P<0.001; F = 455.66), COP_V_ (P<0.001; F = 335.86) as well as ankle (frontal: P<0.001; F = 90.19; sagittal: P = 0.03; F = 16.55), knee (sagittal: P<0.001; F = 290.16) and hip (frontal: P<0.001; F = 85.69; sagittal: P<0.001; F = 125.24) joint excursions increased with increasing perturbation displacement.

### Velocity of the perturbation

The perturbation velocity affected the early reflex components SLR and MLR, whereas LLR remained unaffected. Muscle activation was scaled to increasing perturbation velocity for the muscles SOL (P<0.001; F = 71.08), GM (P = 0.04; F = 4.81) and PER (P = 0.04; F = 7.08) as well as for Gmed (P = 0.02; F = 16.31) in SLR, and for the shank muscles SOL (P<0.001; F = 126.38), GM (P<0.001; F = 109.25), TA (P = 0.02; F = 17.26) and PER (P<0.001; F = 58.13) also in MLR (Fig 3B).

COP_V_ (P<0.001; F = 82.85) increased with increasing perturbation velocity, whereas COP_D_ (P<0.001; F = 66.36) and joint excursions in ankle (frontal: P = 0.01; F = 20.14; sagittal: P = 0.04; F = 15.84), knee (sagittal: P<0.001; F = 130.56) and hip (frontal: P = 0.02; F = 10.94; sagittal: P = 0.03; F = 16.39) joints decreased.

### Interactions

The ANOVA revealed significant interaction effects ***(displacement x velocity)*** for the shank muscles PER (P = 0.02; F = 6.07) in SLR, for GM (P = 0.02; F = 6.44), SOL (P<0.001; F = 17.88) and Gmed (P = 0.04; F = 5.08) in MLR, and for TA (P<0.001; F = 34.87) and GM (P = 0.01; F = 8.65) in LLR; increased perturbation velocity significantly facilitated the effect of an increase in perturbation displacement on EMG activity. For the thigh muscles RF (P = 0.005; F = 10.12), VL (P = 0.009; F = 8.35) and BF (P = 0.03; F = 5.64) significant interaction effects ***(displacement x velocity)*** occurred delayed only in LLR (Table 2, Fig 3B). Accordingly, significant interaction effects ***(displacement x velocity)*** were observed for COP_D_ (P<0.001; F = 52.18), COP_V_ (P = 0.002; F = 13.66), ankle joint excursions in the frontal (P<0.001; F = 24.45) and knee joint excursions in the sagittal plane (P = 0.02; F = 6.75), indicating a distinct interrelation in kinematics with increasing velocity in response to increased displacement.

Further, the ANOVA revealed significant interaction effects for COP_D_ ***(direction x displacement***: P<0.001; F = 99.28 and ***direction x velocity*:** P = 0.02; F = 7.46) and for COP_V_ (***direction x displacement***: P<0.001; F = 70.64 and ***direction x velocity*:** P<0.001; F = 23.48): Increasing perturbation displacement and velocity revealed greater deflections in a-p (sagittal plane) than in m-l (frontal plane) direction.

The significant interaction effects (***direction x velocity)*** for hip joint excursions in the frontal (P<0.001; F = 23.91) and for ankle joint excursion in the sagittal plane (P = 0.01; F = 7.51) and ***(direction x displacement)*** for ankle joint excursions in the frontal (P<0.001; F = 17.86) and for knee joint excursions in the sagittal plane (P<0.001; F = 25.08) indicating that changes in response to increasing displacement or velocity were differently allocated throughout the limb segments dependent on the direction of perturbation (Fig 2).

The interaction effects between all perturbation determinants ***(direction x displacement x velocity)*** were observed with respect to COP_D_ (P = 0.02; F = 3.44) and COP_V_ (P = 0.03; F = 3.95): Analyses revealed that the augmented responses to increasing perturbation displacement during the faster velocity were more pronounced in the sagittal than in the frontal plane. Moreover, the interaction effect between all perturbation determinants ***(direction x displacement x velocity)*** was observed with respect to ankle (P = 0.001; F = 14.16) and hip (P = 0.02; F = 6.23) joint excursions in the frontal plane, indicating dependencies of the directions (medial or lateral) among the two displacements and velocities.

## Discussion

The objective of this study was to assess the interaction effect of three perturbation determinants on postural neuromuscular and kinematic responses while balancing in unilateral stance. The study revealed four main results: (1) early reflex components (SLR and MLR) were scaled to increasing velocity, whereas the later component (LLR) was scaled to increasing displacement. (2) Moreover, phasic assignments to increasing velocity or displacement of the perturbation also revealed segmental preferences to regain balance using distal muscles for fast compensation in SLR and proximal muscles to stabilise in LLR. (3) Further, kinematic distinctions regarding the compensation for balance disturbances indicated plane- and segment-specific dependencies with respect to perturbation displacement and velocity. (4) Velocity is suggested to be the key parameter that significantly facilitates the effect of the other parameters, particularly on neuromuscular activation.

### Main effects

There are two aspects elucidated through the main effects:

i Neuromuscular compensation to changes in velocity of the perturbation occurred predominantly in SLR and MLR. Particularly the short latency compensation of balance disturbance was only present during high velocity perturbations (e.g. SOL, GM, TA, PER, see Table 2). As known from literature, muscle activity during SLR is commonly not observed during translational perturbations; however, when the velocity is sufficiently high a small SLR is visible. Modulations in muscle activity during SLR are attributed to the spinal input of Ia afferent fibres containing the monosynaptic reflex [5,26,34–37], whereas the functionally relevant MLR [2,3,17] is supposed to be modulated by supraspinal structures via polysynaptic pathways of group II afferents [2,17,35,36,38–40]. Sensory information transmitted via Ia and II afferent reflex circuits are related to the velocity-sensitive muscle spindle to immediately counteract increased perturbation velocity [3,5]. There is evidence that the ability to detect stimulus velocity instantaneously [5,38] is attributed to the high conductibility of the Ia afferent fibres, which enables muscle spindle receptors to deliver fast information needed for corrective responses [37,41]. In contrast to velocity-induced changes, the effect of increasing perturbation displacement revealed phase-specific compensation only in LLR, which is supposed to involve direct corticospinal pathways [5,26,36,38,42,43]. As it is reported that the SLR is not sensitive to changes in displacement and hence was only present after the fastest perturbations in some muscles, the late component LLR may be necessary to compensate for substantial balance disturbances [3,5,43].

Functional consequences of the phase-specific differences in displacement- and velocity-induced neuromuscular responses are reflected by modulated joint kinematics and COP displacement; fast compensation in SLR may provide an appropriate torque in the ankle joint to counteract the perturbation and regain balance at an early stage [44]. Thus, although velocity was enhanced, joint excursions and COP displacement remained unchanged or even decreased. Delayed compensation on the neuromuscular level, however,–as it is observed in LLR for enhanced perturbation displacement–caused increased kinematic reactions, i.e. augmented joint deflections and enhanced COP displacement and velocity, associated with significant difficulties to regain postural equilibrium [45], for instance also observed after knee surgery [46].

ii The second aspect deals with leg segments: The abovementioned phase-specific compensation is reflected in distinct postural strategies involving specific segments of the limb. For compensation of augmented perturbation velocity, the shank muscles are of considerable importance to regain equilibrium, while proximal muscles are barely involved (Fig 3B). According to literature, quickly delivered reflex activations in distal muscle groups are linked to stretch velocity and, thus, provide appropriate and fast isolated distal joint torques to restore balance [21]. Hence, ankle joint stiffness is increased, leading to less joint excursions and COP displacement. In contrast, our study revealed that both shank and thigh muscles were activated to regain equilibrium when perturbation displacement was increased (Fig 3B). It is suggested that the late reaction in LLR required a recruitment of the proximal muscles in addition to the distal muscles [3,5,21]. Consequently, an increase in displacement caused an overall increase in kinematics involving the proximal segments reflected in larger COP displacements and knee and hip joint deflections.

### Interaction effects

There are three aspects elucidated through the interaction effects:

1. Reflex phases and limb segments: Our findings suggest that the timing of the neuromuscular response to both increasing displacement and velocity was different for the distal and proximal limb. Compensatory muscle activity of the shank muscles occurred in SLR, whereas the majority of thigh muscles contributed to balance recovery only in LLR (Fig 3B). This observation seems to be largely determined by the anatomical properties and function of the target muscles—distal muscles acting on joints near to and proximal muscles stabilising joints far from the postural disturbance [3,19]. According to Moore et al. [19], the distal limb segment is related to platform velocity and thus, muscles may serve as “prime movers” to produce fast corrective responses around the ankle joint. Conversely, containing delayed EMG bursts in LLR, the proximal muscles are supposed to act as “stabilisers” to compensate for the resulting torque transferred between limb segments after distal muscle activity [3,19]. In view of the latter aspect, our results detected different phase- and segment-specific neuromuscular strategies between the distal and proximal limb, finely attuned to the augmented balance disturbance.
2. Plane- and segment-specific kinematic interrelations: Interactions elucidated direction-specific effects of displacement and velocity on kinematic strategy. While both–increased displacement and velocity–were compensated throughout increasing deflections in the ankle joint in all directions, we observed direction-specific distinctions in the knee and hip joint. In the sagittal plane, predominantly knee joint deflections compensated for displacement-induced balance disturbance, pointing towards distal regulation of balance recovery in a-p direction [22] (Fig 2). This observation is supposed to be attributed to the functional range of motion of the ankle and knee joints, which enable the body to lower the COG height leading to a rapid reacquisition of a stable COG state during unpredictable slips by deflecting the respective joints [47–49]. Contrarily, the hip joint gained importance for equilibrium recovery when perturbations were applied in the frontal plane (Fig 2). As we conducted the measurements in a single instead of both leg stance, the support surface is considerably smaller in the frontal plane and may lead to a considerably increased postural demand. In particular in the frontal plane, parallel feet position of bipedal stance secures equilibrium by shifting the load from one foot to the other, which is mechanically impossible to execute in unilateral stance. Interactions revealed that hip joint deflections were particularly sensitive to velocity-induced changes, predominantly in m-l perturbations. According to literature, the proximal regulation provides evidence for the use of a hip strategy to properly adjust the COG above the base of support when postural tasks are more challenging, as it occurs throughout increasing velocity [8]. Interlinked with the EMG data, we suggest that, by controlling a multi-segment system, kinematic control after balance perturbation includes a segmental distribution of compensatory responses (Fig 3B). As the base of support does not restrict the subjects freedom of movement, hardware constrains are excluded to be responsible for the observed differences.
3. Key parameter velocity: Interactions elucidate perturbation velocity to be the target parameter, which predominantly facilitates the effect of the other determinants. As changes in velocity considerably influenced early reflexive muscle activation detected in the SLR and MLR [16], our results indicate that displacement-induced adaptations, mainly visible in the LLR, are influenced by the extent of the velocity as well. As a major consequence, neuromuscular compensation due to increased displacement showed gradually elevated activation, however, and most importantly, those effects were only detected during the fast velocity condition, whereas displacement-induced changes during the slow velocity condition remained mostly unaffected. Aforementioned aspects indicate a significant increase in postural demand associated with considerably elevated postural reactions during increased velocity.

### Limitation

Although acceleration and deceleration profiles are coded in the displacement and velocity of the perturbation, they may have an additional effect on the output of postural responses [13]. It is assumed that the higher the displacement and velocity of the perturbation, the higher the acceleration and deceleration profile of the platform movement and the bigger the postural response. However, the amount of 320 perturbations needed for an assessment of the three parameters direction, displacement and velocity with their characteristics did not make it possible to control for two more parameters. Consequently, there is a need for further investigations to clearly assess effects and interactions of acceleration and deceleration profiles with other variables.

## Conclusion

This study provided new insights on the neurophysiological and kinematic regulation of postural responses during unilateral balancing by applying random, unexpected perturbations. This is considered to be a challenging postural task which requires appropriate neuromuscular control to regain equilibrium after surface translation [1], as it is needed during slip-like conditions. For the first time, reactive balance control was examined during unilateral balance tasks assessing how the different perturbation determinants interact and how these interactions are represented in the postural response. Main and interaction effects indicate that compensatory postural responses are based on complex processes that include different postural strategies characterised by phasic and segmental specifications, precisely adjusted to the respective type of balance disturbance.
